# Chromatin Dynamics in Lineage Commitment and Cellular Reprogramming

**DOI:** 10.3390/genes6030641

**Published:** 2015-07-17

**Authors:** Virlana M. Shchuka, Nakisa Malek-Gilani, Gurdeep Singh, Lida Langroudi, Navroop K. Dhaliwal, Sakthi D. Moorthy, Scott Davidson, Neil N. Macpherson, Jennifer A. Mitchell

**Affiliations:** Department of Cell and Systems Biology, University of Toronto, Toronto, ON M5S 3G5, Canada; E-Mails: virlana.shchuka@mail.utoronto.ca (V.M.S.); nakisa.malek.gilani@mail.utoronto.ca (N.M.-G.); gur.singh@mail.utoronto.ca (G.S.); lida.langroudi@mail.utoronto.ca (L.L.); navroop.dhaliwal@mail.utoronto.ca (N.K.D.); sakthi.moorthy@utoronto.ca (S.D.M.); s.davidson@utoronto.ca (S.D.); neil.macpherson@utoronto.ca (N.N.M.)

**Keywords:** embryonic stem cell, induced pluripotent stem cell, reprogramming, chromatin, epigenetics, chromatin looping, transcription factor, gene expression, differentiation

## Abstract

Dynamic structural properties of chromatin play an essential role in defining cell identity and function. Transcription factors and chromatin modifiers establish and maintain cell states through alteration of DNA accessibility and histone modifications. This activity is focused at both gene-proximal promoter regions and distally located regulatory elements. In the three-dimensional space of the nucleus, distal elements are localized in close physical proximity to the gene-proximal regulatory sequences through the formation of chromatin loops. These looping features in the genome are highly dynamic as embryonic stem cells differentiate and commit to specific lineages, and throughout reprogramming as differentiated cells reacquire pluripotency. Identifying these functional distal regulatory regions in the genome provides insight into the regulatory processes governing early mammalian development and guidance for improving the protocols that generate induced pluripotent cells.

## 1. Introduction

Early mammalian development is a process that progressively alters cell plasticity as cells commit to their differentiated state. These changes in cell state are accompanied by dynamic changes in the transcriptome, chromatin properties and nuclear organization of the genome. In the early embryo, cells of the inner cell mass (ICM), and later of the epiblast, are pluripotent. These cells differentiate into cells of the three germ layers, endoderm, mesoderm, and ectoderm, which give rise to all cells of the mature organism. These cells are also capable of self-renewal, as demonstrated by embryonic stem (ES) cells and epiblast stem cells (EpiSC), which are isolated from the early embryo, are maintained in culture in the pluripotent state and can be induced to differentiate (*in vitro* or *in vivo*) into cells from each of the three germ layers [1,2,3].

Dynamic changes in the transcriptome that occur during lineage commitment and cellular reprogramming are orchestrated by *cis*-regulatory elements and *trans*-regulatory factors. The *cis*-regulatory elements (CREs) are regions that control transcription such as promoters, enhancers and insulators. While promoters are immediately upstream of the gene transcription start sites (TSS), enhancers and insulators can be located at megabase (Mb) distances from the TSS of the gene(s) they regulate. These distal CREs form chromatin loops to contact and regulate their target genes; as a result, there has been an increasing interest in understanding chromatin folding properties in different cellular contexts. CREs are sequences that function through binding of transcription factors and co-factors, the *trans*-regulatory factors (TRFs), which in turn regulate RNA polymerase association with DNA and its activity. Chromatin accessibility and transcriptional output from the genome depends on TRF availability and activity. TRFs combinatorially establish and maintain cell states, as revealed by the observation that ectopic expression of transcription factors alters cell state. Pioneered by Takahashi and Yamanaka, the cell reprogramming experimental approach involves expression of four ES cell-expressed transcription factors, *Oct4* (also known as *Pou5f1*), *Sox2*, *Klf4*, and c-*Myc* (OSKM), to reprogram somatic cells to an induced pluripotent stem (iPS) cell state [4].

In addition to the changes in chromatin accessibility and transcriptional output throughout lineage commitment and cellular reprogramming, interphase nuclei display a dynamic structural reorganization of their genomes. The folding patterns adopted by a cell’s genome in three-dimensional nuclear space are critical for establishing cell identity and maintenance of the transcriptional program. Across cell types, differential chromosome conformations reflect a complex hierarchical compartmentalization of the genome. Chromosomes occupy largely discrete regions within the nucleus known as territories [5]. Individual chromosome territories are further sub-divided into Mb-sized topologically associated domains (TADs) [6]. TADs in turn contain contact domains less than 200 kilobases (kb) in size [7] and domains housing chromatin loops of various sizes. Many such chromatin loops are highly cell type-specific and allow CREs otherwise distal to one another in the linear genome to be brought into close spatial proximity to a gene TSS, an event associated with that gene’s expression. While other models of enhancer function that involve either partial or no loop formation have been proposed [8], several genome folding studies highlight the role of chromatin loop formation in gene regulation.

Repositioning of gene loci within the nuclear space and altered configuration of entire chromosomes occur as ES cells differentiate and somatic cells undergo reprogramming. Despite these changes, some architectural features of genome organization appear to be more universal and are conserved throughout cellular differentiation. In this review, we discuss the dynamic features of chromatin and genome topology in the context of lineage commitment and cellular reprogramming and highlight emerging mechanisms controlling the concomitant changes in cellular phenotypes.

## 2. Transcriptional Control of Lineage Commitment and Reprogramming

A large number of transcription factors with lineage-specific expression patterns in the pre-implantation embryo have been identified. Many such factors are required for pluripotency and for one or more of lineage establishment, maintenance or differentiation. In the early embryo the HIPPO signaling pathway is the earliest identified signaling mechanism; this pathway requires the TEAD transcription factor family member, *Tead4*. Mouse mutants lacking *Tead4* die prior to the blastocyst stage due to a failure to form trophectoderm, which consists of cells that differentiate to extra-embryonic tissues like the placenta [9,10]. At the same time the HIPPO pathway restricts *Sox2* expression to ICM progenitors prior to the blastocyst stage [11]. *Sox2* null embryos develop past the blastocyst stage but die shortly after implantation due to a failure in maintaining pluripotent epiblast cells [12].

The OCT4 transcription factor, which binds DNA as a dimer with SOX2 to regulate transcription, is also required for pluripotency maintenance in the early embryo [13,14,15]. *Oct4* deleted embryos die prior to implantation due to an inability to maintain pluripotency in the ICM, and cells of the ICM are instead restricted to the trophectoderm lineage [15]. In the mouse 8 cell embryo, fluorescence decay after photoactivation (FDAP) has been applied to determine the binding kinetics of pluripotency-associated transcription factors [16]. Before other morphological signs of lineage commitment can be observed, OCT4 displays slower kinetics in cells that later commit to the ICM lineage compared to those that contribute to the extra-embryonic lineage. Additionally, both OCT4 and SOX2 exhibit slower dynamics in the established ICM than in the trophectoderm [17].

Although not one of the original Yamanaka factors, NANOG is also involved in maintaining pluripotency through binding of CREs in conjunction with OCT4 and SOX2 [14]. Homozygous deletion of *Nanog* causes pre-implantation lethality in mice; in these embryos the ICM forms but loses pluripotency and later forms only parietal endoderm-like cells [18]. Furthermore, *Nanog* over-expression in ES cells negates the need for LIF (Leukemia Inhibitory Factor) in culture media, revealing that *Nanog* expression can maintain pluripotency in the absence of external stimuli [18]. In addition to their involvement in maintaining pluripotency in ES cells and in cells of the early embryo, many of these transcription factors are also required for early lineage commitment [19].

As previously mentioned, reprogramming somatic cells to iPS cells was first accomplished by forced expression of a cocktail of four transcription factors, OSKM. These transcription factors modify the epigenome in the somatic cell, causing genome-wide opening of chromatin, and eventually establish the transcriptome in iPS cells [20]. During the early stages of reprogramming, stochastic gene expression occurs in the entire cell population and only a very small subset will eventually become true iPS cells [21]. Select genes, such as *Esrrb* and *Dppa2*, are predictive markers for cells that are likely to progress to pluripotency. During later reprogramming stages expression of endogenous *Sox2* is highly deterministic of the few cells that will form fully reprogrammed iPS cells [21]. This is not the case for all reprogramming factors; expression of endogenous *Oct4* does not specifically mark cells that will go on to form iPS cells as many *Oct4*-positive cells stall at an intermediate partially differentiated stage [21]. Since reprogramming with OSKM is inefficient, with few cells acquiring a full iPS state, there has been significant focus on optimizing reprogramming procedures.

Multiple routes to reprogramming have been developed through transcription factor substitution or addition to the OSKM cocktail. For example, *Esrrb* can replace *Klf4,* and *Lin28* can replace c-*Myc* in the OSKM cocktail [22,23]. Similarly, functional redundancy between members of the same protein family exists; *Klf2* and *Klf5*, both of which are expressed in ES cells, are redundant with *Klf4* in the core ES cell transcriptional circuitry [22]. All GATA family proteins are capable of replacing *Oct4* in reprogramming and are thought to do so by supporting expression of *Sall4*, another essential pluripotency-associated transcription factor [24]. Not all pluripotency factors, however, are required for reprogramming, as *Nanog* is dispensable for iPS cell generation [25]. *Nanog* null fibroblasts can give rise to *Nanog* null iPS cells whose transcriptome is highly similar to that of wild type iPS cells. Regardless of the cocktail of reprogramming factors used, these proteins must alter the epigenetic landscape of the differentiated cell to establish pluripotency.

## 3. Dynamic Chromatin Modifications

Observations of whole nuclei reveal that the chromatin landscape of ES cells is a loosely packed mesh of fibers, with less condensed chromatin than that observed in somatic cells [26]. Additionally, super-resolution microscopy indicates that nucleosomes are found in clusters throughout the nucleus, with ES cells having smaller and less dense clusters than differentiating cells, which reflects a more open chromatin configuration in ES cells [27]. In the tightly associated arrangement found in heterochromatin, histone H1 is bound at the linker DNA between nucleosomes. Euchromatic regions containing expressed genes are more loosely associated with nucleosomes and display unbound linker DNA between individual nucleosomes [28].

The degree to which DNA is exposed for regulation depends greatly on epigenetic modification of the histones, particularly histone H3. These modifications include acetylation (ac) and mono-, di- or tri-methylation (me1, me2, me3) of lysine residues, with functions that differ depending on the nucleosome subunit and specific residue that has been modified. Furthermore, chromatin is remodeled by proteins that slide and remove nucleosomes and create nucleosome-free regions at active gene TSS and distal CREs. A region’s specific patterns of histone modifications can be diagnostic of the potential activity of that region. For example, H3K36me3 is associated with transcribed gene bodies, whereas the TSS of active genes are marked by H3K4me3 [29,30,31]. Different H3K27 methylation states denote distinct regions that do not overlap; H3K27me1 is found in intragenic transcribed regions and correlates with H3K36me3 deposition, whereas H3K27me2 is found across broad intergenic and intragenic regions [32]. H3K27me3 by contrast is associated with Polycomb-mediated gene repression in mammalian cells [33,34]. H3K9me3 is associated with heterochromatic regions of the genome that, in ES cells and fully reprogrammed iPS cells, are organized in accessible 10 nm chromatin fibers similar to those in the surrounding euchromatin [35,36]. In somatic and partially reprogrammed cells, H3K9me3-marked nucleosomes are found in tightly packed chromocentres that are not observed in ES cells [36]. Interestingly, in pluripotent cells, a bivalent state has been observed in which regions are modified by both H3K27me3 and H3K4me3; this state is associated with inactive, poised developmentally regulated genes that become active as the cells differentiate [36]. Furthermore, a group of regulatory regions distinct from poised enhancers has been identified in select differentiated cells [37]. Dubbed latent enhancers, these regions are bound by transcription factors and acquire active H3K4me1 and H3K27ac marks only upon cells’ exposure to biochemical stimuli. Removal of said stimuli results in a concomitant depletion of H3K27ac and regulator occupancy without affecting H3K4me1 enrichment. Later re-stimulation causes a faster and stronger response at latent enhancers than that observed during the first stimulus exposure, an observation consistent with the notion of H3K4me1 retention facilitating the re-activation of these regulatory regions [37]. The identification of poised and latent enhancers therefore highlights the dynamic nature of histone modifications. 

Changes in cell states are accompanied by profound changes in the transcriptome of the cell, which in turn arise from changes in the activity of transcriptional regulators at CREs. Studies investigating chromatin modifications in different cell types revealed that promoter modifications were more consistent across different cell types whereas distal regions displayed more dynamic cell type-specific modifications [38]. Some of these distal regions display enhancer activity, or position- and orientation-independent activation of transcription in reporter assays. Putative enhancers defined by ChIP-seq are nucleosome-depleted and have an array of histone marks and protein associations that appear to separate their function into distinct classes. Putative enhancer regions near transcribed genes in ES cells are marked by enrichment of both H3K4me1 [31] and H3K27ac [39], binding of chromatin modifiers p300 and BRG1 [40,41] and binding of multiple transcription factors [14,42]. By contrast, poised enhancers are enriched with H3K27me3, are depleted of H3K27ac and are proximal to inactive genes [41,43,44,45]. Poised enhancers are thought to regulate genes required upon differentiation of ES cells, since they acquire H3K27ac during commitment to specific lineages [41,43,44,45].

Ectopic expression of ES cell-specific transcription factors results in genome-wide epigenetic and transcriptional alterations, allowing cells to move back toward pluripotency [46]. Treated cells that reach the fully reprogrammed state of iPS cells regain their pluripotent characteristics, are capable of forming all three germ layers and can be used to generate viable mice [47]. The early steps of somatic cell reprogramming by expression of OSKM are characterized by depletion of global H3K27me3 [20], mediated by demethylases including KDM6A [48]; re-establishment of global H3K27me3 levels occurs as cells acquire a reprogrammed state [20]. In the early stages of reprogramming, promoters for genes that are expressed later in reprogramming are marked by enhanced H3K4me2 peaks [49]. *Nanog* expression is induced in partially reprogrammed cells [50], and high *Nanog* levels are associated with a loss of compaction at chromocentres, a feature of pluripotent cells [36]. Regions bound by specific TRFs display different dynamics, with OCT4/SOX2, KLF4 and NANOG binding sites losing H3K27me3 early in reprogramming and binding sites for ESRRB and TCFCP2L1 losing H3K27me3 later in reprogramming [51]. All these regions maintain a demethylated state in fully reprogrammed iPS cells.

## 4. Enhancers: Critical Regulatory Elements in Lineage Commitment and Reprogramming

In both pluripotent and differentiating cells, transcriptional regulatory networks are organized by spatiotemporal contact between distal regulatory elements (often enhancers) and their associated promoters to generate cell type-specific gene expression patterns [52]. The coordinated action of critical TRFs binding to specific motif sequences within both promoters and more distal enhancers maintains the ES cell transcriptome [14,53,54]. TRF activity at distal enhancers is critical in modulating gene expression, in guiding cell fate specification during developmental transitions, and in acquiring the iPS cell state during reprogramming [55,56].

Enhancers are often located at significant distances from the genes they regulate, and function in a position- and orientation-independent manner; as a result they have been difficult to identify, functionally characterize, and link to the genes they regulate. ChIP-Seq studies that investigated OCT4, SOX2 and NANOG binding throughout the genome found that regions of ~1 kb co-bound by all three factors in ES cells exhibited enhancer activity in reporter assays [14]. Super-enhancers that cover larger regions (median size 8 kb) and often contain more than one OCT4, SOX2, and NANOG co-bound region, have been proposed to regulate cell identity-associated genes [54,57]. These proposed CREs were defined either by higher association with the mediator complex or increased levels of H3K27ac [54,57]. While there is debate as to the function of super-enhancers throughout the genome [58], one such region, the *Sox2* Control Region (SCR), has been shown to be essential for *Sox2* transcription and maintenance of the embryonic stem cell phenotype [59,60]. Deletion of the SCR in ES cells impairs ES cell differentiation to neuroectoderm, revealing the important role this region plays in early lineage commitment [59]. Many enhancers are thought or known to be required for lineage commitment (Table 1). Transcription factors involved in lineage commitment bind to these enhancers and may modulate gene expression in a lineage-specific way [61,62,63,64].

**Table 1:** Enhancers and their predicted or known role in lineage specification.

Name of Enhancer and/or Its Targeted Gene	Relative Enhancer Location	Predicted or Known Specified Cell Lineage
*Pu.1*	14 kb upstream of the *Pu.1* promoter	Monocytes [65]
*Mpo*	3.2 kb upstream of *Mpo*	Promyelocytes [66]
*Bmp4*	46 kb upstream of *Bmp4* promoter	Mesoderm [67]
SCR, enhancer of *Sox2*	104–112 kb downstream of *Sox2*	Neuroectoderm [59]
HBE, enhancer of *Nodal*	3 kb upstream of *Nodal* TSS	Extra-embryonic precursors [68]
SHF-specific *Isl-1*	4 kb downstream of *Isl-1*	Cardiac progenitors [69]
Enhancers of *Pax5*	5th intron of *Pax5* locus 5' flanking region of *Pax5*	B-lineage lymphocytes[70] MHB boundary [71]
*Myf5*	48 kb upstream of *Myf5* TSS	Epaxial muscle progenitors [72,73]
*Fn1*	12th intron of *Fn1*	Extra-embryonic endoderm [74]
AHF-specific enhancer of *Mef2c*	16.3 kb downstream of the first untranslated *Mef2c* exon	Cardiac progenitors [75]
U2, enhancer of *Sox10*	47.3 kb upstream of *Sox10*	Oligodendrocyte precursors [76,77]
Ectodermal enhancer of *Pax6*	3.5 kb upstream of *Pax6*	Corneal lineage [78]
Distal enhancer of *Pax6*	125 kb downstream of *Pax6*	α-cells and neural lineage [79,80]
Enhancers of *Gata1*	25 kb upstream, 3.5 kb upstream, and 6 kb downstream of first *Gata1* exon erythroid promoter	Erythroid lineage [81]
B108, enhancer of *Blimp1*	11 kb downstream of *Blimp1*	Retinal cells [82]

TSS—transcription start site; AHF—anterior heart field; HBE—highly binding element; SCR—Sox2 control region; SHF—second heart field; MHB—midbrain-hindbrain boundary.

Some transcription factors have pioneer activity, or the ability to access condensed chromatin, alter the accessibility of enhancers, and allow the binding of subsequent factors. Conversely, other transcription factors bind mainly to accessible enhancers and thus require a pre-existing chromatin configuration. The latter is the case for FOX3P, which is required during T-cell lineage specification [83]. Some enhancers for genes involved in orchestrating the early events in lineage commitment therefore need to be epigenetically poised for activation, as previously described [41,84]. These poised enhancers are enriched with H3K4me1 in ES cells and are selectively activated during differentiation whereby they acquire H3K27ac to support the new developmental state [85]. For example, the Neuroglycan-C and Neurophilin-2 loci harbor poised enhancers in ES cells that acquire H3K27ac in neural progenitor (NP) cells [44].

During the initial phase of somatic cell reprogramming, distal regulatory elements located in closed chromatin are bound by OSK, revealing the pioneer activity of this complex [86]. Regulatory elements accessible to OSK despite their closed chromatin state are near genes that are important in the process of reprogramming, such as genes involved in mesenchymal to epithelial transition (MET) [86]. Pioneer activity of the OSK complex, however, is impeded at H3K9me3-enriched regions of the genome, which include genes involved in the later stages of reprogramming, such as *Nanog* and *Sox2* [86]. A subclass of permissive enhancers in fibroblasts are modified by H3K4me1 and nucleosome depletion, and exhibit DNase I hypersensitivity [87]. These permissive enhancers allow binding of ectopically expressed factors regardless of their pioneering potential, and co-ordinate changes to their cognate promoters. For example, ectopically expressed OCT4 binds the *Myod1* enhancer and stimulates acquisition of a bivalent domain at the *Myod1* promoter similar to that observed in ES cells [87]. Conversely, binding of MYOD1 to this same enhancer causes the promoter to acquire an active chromatin signature and leads to subsequent activation of *Myod1* transcription. In these examples, binding of transcription factors to distal regulatory elements precedes changes in chromatin state at the associated promoters, displaying the mechanistic importance of CREs in the reprogramming process. Specific enhancers have also been targeted and activated using designer transcription factors to enhance ES cell conversion to iPS cells, further illustrating the importance of these regions in the reprogramming process [88,89].

## 5. Genome Architectural Changes during ES Cell Differentiation

As with smaller scale chromatin features, entire chromosomes exhibit specific structural properties dependent upon cell type. The ES cell nucleus has unique features that are associated specifically with pluripotency and are thought to be required for the balance between self-renewal and differentiation. ES cells have been shown to be rich in euchromatin [90], perhaps in part due to the abundant expression of proteins involved in chromatin remodeling [91]. Coupled with elevated levels of euchromatin, ES cell nuclei display increased global transcription levels [92] and approximately twice the total number of sites of nascent transcription, termed transcription factories, compared with differentiated cells [93]; however, transcription factory density and diameter are similar between ES and differentiated cells. Although not conducted in pluripotent cells, the application of live-imaging techniques to several primary cell lines has verified the existence of transcription factories in live cells [94,95]. The observations of open chromatin state and elevated gene expression are consistent with those of highly plastic chromatin displaying more dynamic movements in the ES cell nucleus prior to differentiation [96,97]. Increased global transcription and chromatin remodeling protein expression were shown to be altered upon differentiation. Furthermore, the differentiation process induces genome compaction, increasing the proportion of heterochromatin in the nucleus [98]. The spatial organization of chromosomes within the nucleus has also been demonstrated to be cell type-specific [99]. Fluorescence *in situ* hybridization (FISH) experiments have shown that entire chromosomes occupy distinct territories in the nucleus. The relative positions of individual chromosomes display cell type-associated preferences [5,100]. One well studied differentiation-associated chromosome re-arrangement is the inactivation of one of the two X-chromosomes in female cells. A molecular event that ensures correct expression dosage of X-linked gene products, this inactivation is thought to be achieved by the three-dimensional spreading of *Xist*, which mediates the compaction of one X chromosome through recruitment of the polycomb complex PRC2 as naïve ES cells differentiate toward EpiSC [101]. Interestingly, reversal of X chromosome inactivation through the removal of *Xist* and alteration of the epigenome during reprogramming requires a direct reversal of the inactivation process to achieve pluripotency, and can only be brought about through the expression of pluripotency-associated genes [102].

Changes in chromosome structure can also be observed by chromosome conformation capture (3C), a molecular technique that captures proximity between chromatin regions through formaldehyde cross-linking and *de novo* ligation of chromatin fragments contained within the same cross-linked chromatin complex [103]. Chromosome architecture has been modeled from a high throughput 3C variant, Hi-C, which revealed that the genome resembles a fractal globule structure [104] that theoretically allows for dynamic rearrangements without spatial conflicts such as knotting. These Hi-C experiments support other studies’ observations that some genes “loop out” of their chromosome territories upon activation [105] and contact genes from other chromosomes at transcription compartments [106,107]. In ES cells, exonic regions are commonly found near the periphery of their respective chromosome territories, perhaps due to the higher global transcriptional activity and increase in the proportion of euchromatin observed in the nucleus [90,108]. Hi-C has provided a higher resolution analysis of chromatin architecture in ES cells, revealing the aforementioned Mb-scale, self-preferential interacting TAD regions [6]. Unlike some other architectural features of the genome in ES cell nuclei, these domain boundaries were found to be largely stable across cell types and species. TAD boundaries are enriched in the architectural protein CTCF, a protein that binds insulator regions [6]. TAD domains have also been linked to replication domain boundaries and correlate with the replication timing of euchromatin and heterochromatin in ES cells [109]. The conservation in genome organization at the TAD scale suggests that ES cell differentiation does not disturb, or require the reorganization of, TAD boundaries.

## 6. Dynamics of Chromatin Looping within TADs

Chromatin loops directly implicated in gene regulation generally reside within TAD boundaries. Many of these smaller scale genome folding events are chromatin interactions that connect enhancers or putative enhancers to TSS. Distances between distal CREs and target gene promoters vary but can be in the Mb range. Studies testing for the presence of putative enhancer-TSS contacts at a single locus level have used the traditional 3C approach previously mentioned [103] or the 3C-based variant 4C [110]. Interrogation of genome-wide putative enhancer-TSS chromatin loops has involved several genome-wide or high throughput variants of 3C, which include 5C [111], ChIA-PET [112], Hi-C [104], Capture-C [113] and Capture Hi-C [114].

Both regulatory and constitutive genome-genome contacts appear to be supported by one or more of the following: the aforementioned TAD boundary-associated CTCF, the cohesin complex and the mediator complex. At the loci of several genes integral for ES cell function, most chromatin interacting regions are enriched by binding of the cohesin complex subunit SMC1 or co-binding of SMC1 with either CTCF or mediator [53]. Consistent with the critical role of chromatin loops in stem cell maintenance, these three proteins are all necessary for ES cell sustainment [115,116]. The loop-associated protein list has also been extended to include ZNF143, a human ES cell identity determinant [117] and a protein that co-occupies CTCF- and SMC1-bound loop regions in various cancer cell lines [7,118,119]. The murine equivalent ZFP143 is indispensable for *Nanog* regulation and ES cell self-renewal [120], which further aligns its function and action mechanism in ES cells to those of the more extensively studied loop-associated proteins. Some combination of these proteins binds the majority of chromatin interaction anchor sites in ES cells, but only a quarter of the anchor sites in NP cells [53]. This observation suggests that as yet uncharacterized or undiscovered factors may regulate and support loop formation in a lineage-specific manner.

Less is known regarding the putative role of pluripotency factors in the establishment of ES cell-specific chromatin loops. The enrichment of tissue-specific SMC1 binding at ES cell-specific chromatin interaction sites in either the presence or absence of OCT4, SOX2, and NANOG co-binding initially suggested that pluripotency factors may be dispensable for the loop anchoring mechanism that is mediated by the cohesin complex [53]. Other studies, however, demonstrate direct dependencies between loop-associated proteins and pluripotency factors. For instance, ZFP143 recruits OCT4 to the *Nanog* promoter, which is bound by both TRFs in ES cells [120]. *Klf4* knockdown reduces both SMC1 binding at the *Oct4* distal enhancer as well as *Oct4* distal enhancer-promoter loop formation in pluripotent stem cells [121]. Furthermore, select TRFs have been implicated in working with known loop-associated proteins to re-configure the chromatin architecture of particular loci in different cell types, such as the TFIID complex subunit TAF3 and SALL4 in endoderm lineage commitment [122,123]. These studies collectively suggest that pluripotency factors may have a role in anchoring the regulatory loops of at least some loci upon cell fate specification.

Studies that interrogate changes in genome folding as ES cells differentiate into cells of disparate lineages have observed instances where chromatin loops can be conserved or can vary across cell types. Both ES cells and NP cells were shown to have overlapping CTCF- and cohesin-bound sites involved in Mb-sized constitutive interactions consistent with TAD boundaries. In contrast, sites co-bound by cohesin and mediator were prevalent in sub-Mb-sized interactions present only in ES cells [53]. At the *Olig1/2* locus, for instance, several CTCF- and cohesin-bound loops whose contact sites flank *Olig1* and *Olig2* in the linearized view of the locus are conserved upon ES cell differentiation to NP cells. However, a smaller ES cell-specific looping event enriched in mediator and cohesin binding was detected between a putative ES cell-specific *Olig1* enhancer and its promoter. Differential *trans* chromosomal interactions were also observed between the *Olig1* and *Olig2* promoters and genes implicated in neuronal development in neural stem cells and NP cells [124], supporting the link between genome folding patterns and cell identity. It has furthermore been postulated that, in addition to establishing TAD boundaries, constitutive interactions may pre-mark differentiated cell-specific regulatory interactions [53]. For instance, the CTCF-, cohesin-, and mediator-enriched SCR region over 100 kb downstream of *Sox2*, loops to the *Sox2* promoter in ES cells, but not in NP cells [53,59]; however, a more distal region bound by CTCF and cohesin in ES cells contacts the *Sox2* promoter in NP cells and is thought to become a NP cell-specific *Sox2* enhancer [53].

The significance of genome folding patterns in determining cell fate is further highlighted by their role in cellular reprogramming. Proper chromatin looping events must be established for successful pluripotency induction in differentiated cells [125]. As is the case for ES cells, cohesin and mediator are indispensable for iPS cell enhancer-promoter contacts and self-renewal [126,127]. Furthermore, regions involved in looping at the *Oct4* and *Nanog* loci are largely shared between ES and iPS cells, but are not observed in fibroblasts [126,127]. Several studies also promote the idea of enhancer activation occurring prior to gene activation. For instance, pluripotent cell-specific chromatin interactions are established at the *Nanog*, *Lefty1* and *Oct4* loci during the early stages of reprogramming and well in advance of gene expression [126]. These results support a reprogramming model in which differentiated cells reacquire an ES cell-specific chromatin architecture that precedes the loop-dependent activation of pluripotency-associated gene expression.

## 7. Conclusions and Future Directions

**Figure 1:**
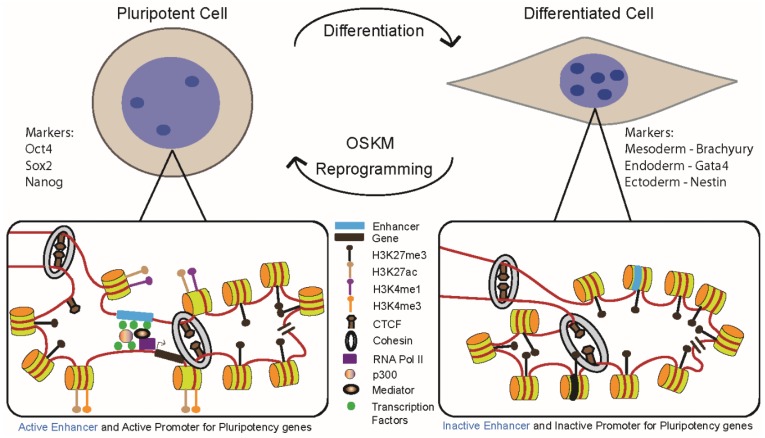
Changes in chromatin accessibility and modification drive the processes of differentiation and reprogramming. Pluripotent cells possess less compact chromatin compared to differentiated cells (light and medium intensity blue, respectively) and lack the compact chromocentres (dark blue) present in differentiated cells (top). The more open chromatin configuration in ES and iPS cells creates less nucleosome compaction (bottom left); as a result, nucleosomes are found in smaller clutches in ES and iPS cells as indicated by two clutches of four and seven nucleosomes, respectively. On the right the more compact chromatin of differentiated cells displays a greater overall density of nucleosomes and larger clutches of nucleosomes. Despite this chromatin compaction in differentiated cells, the reprogramming factors, OSKM, can access enhancer regions in condensed chromatin (blue bar), initiate chromatin remodeling at the enhancer, lead to chromatin remodeling at the gene (black bar) TSS, and eventually activate target genes as cells acquire the iPS state. This process converts the heterochromatic regions in fibroblasts marked by H4K27me3 to euchromatic regions in the iPS cells marked by H3K27ac and H3K4me1 or H3K4me3 at the enhancer and promoter respectively. CTCF and cohesin are found at both tissue-specific and constitutive chromatin loops. The coactivators (p300 and the mediator complex) are found at tissue-specific enhancer-promoter chromatin loops and are thought to bridge transcription factors at distal enhancers to the RNA polymerase II complex at the gene TSS.

Multiple studies have provided evidence that cell lineage commitment and pluripotency induction depend on the structural and functional contributions of CREs and TRFs to cell transcription programs. A model of chromatin dynamics-mediated expression regulation is emerging in which distal enhancer regions play a central role in cell fate specification and reprogramming (Figure 1). Nevertheless, the precise molecular mechanisms by which these elements carry out their regulatory roles and act at a distance with specificity have yet to be determined. Genome-wide dynamic profiling of TRFs and histone modifications throughout the reprogramming process provide a starting point for the identification of putative regulatory regions that drive pluripotency acquisition and maintenance. However, transcription factor regulatory networks are highly interconnected; therefore, the contributions of each transcription factor to the regulation of cell state-maintaining genes and the specific sequence motifs through which these factors function can be difficult to elucidate. Additionally, the identification of all critical transcription factor complex members, as well as the dosage-dependent effects of each factor on the pluripotency transcriptional program, are questions that require further investigation.

In the case of CREs, probing of ES and iPS cell *cis* regulomes by genome-wide chromatin loop-detecting methods has provided a repertoire of putative cell-specific CREs and the gene promoters they contact. The vast majority of these data, however, have emerged from studies of whole cell populations. Additional employment of single cell 3C variants, such as the single cell version of the Hi-C method [128] may provide a more accurate representation of structural chromatin dynamics. Furthermore, the mechanisms through which individual CREs select a cell’s particular lineage commitment pathway are largely unknown. What contributions individual CREs make during the various stages of differentiation into each cell type throughout organism development also remain to be determined. These questions can be more efficiently addressed with the advent of CRISPR-Cas9-based genome editing, CRISPR interference/activation and Cas9-mediated epigenome control [129,130,131,132,133]. Use of these technologies, coupled with various high-resolution and single cell chromatin loop-detecting assays, will provide a better understanding of the players involved in transcriptional regulatory mechanisms.

The need for chromatin dynamics-focused studies is highlighted by the fact that gained knowledge can give rise to new avenues of research with potential for therapeutic developments. For instance, the induction of an artificial chromatin loop between embryonic fetal *globin* and the well-characterized *globin* locus control region (LCR) in both mouse and human erythroid cells has emerged as the basis for potential mitigation of sickle cell anemia and β-thalassemia [134]. This seminal paper, however, has come about as a result of a thorough (and still ongoing) study of the individual molecular players involved in the epigenomic state, genome folding patterns, and transcription activation events at the β*-globin* locus. This demonstrates the need to better characterize the regulatory mechanisms of enhancers at other loci in different cell types. Indeed, similar experiments have enormous potential in the contexts of reprogramming and stem cell therapies. Impediments to the use of reprogrammed cells in a clinical context, including low reprogramming efficiency, extensive required time span, and uncertainty of acquiring the desired differentiated cell state may arise at least in part due to the inability of reprogramming factors to efficiently access and remodel specific enhancers, as described above. Using enhancer activation at these resistant enhancers in pluripotency-induced cells could therefore greatly improve the efficiency of the reprogramming process.

## References

[1] Evans M.J., Kaufman M.H. (1981). Establishment in culture of pluripotential cells from mouse embryos. Nature.

[2] Huang Y., Osorno R., Tsakiridis A., Wilson V. (2012). *In vivo* differentiation potential of epiblast stem cells revealed by chimeric embryo formation. Cell Rep..

[3] Joo J.Y., Choi H.W., Kim M.J., Zaehres H., Tapia N., Stehling M., Jung K.S., Do J.T., Scholer H.R. (2014). Establishment of a primed pluripotent epiblast stem cell in FGF4-based conditions. Sci. Rep..

[4] Takahashi K., Yamanaka S. (2006). Induction of pluripotent stem cells from mouse embryonic and adult fibroblast cultures by defined factors. Cell.

[5] Cremer T., Cremer C. (2001). Chromosome territories, nuclear architecture and gene regulation in mammalian cells. Nat. Rev. Genet..

[6] Dixon J.R., Selvaraj S., Yue F., Kim A., Li Y., Shen Y., Hu M., Liu J.S., Ren B. (2012). Topological domains in mammalian genomes identified by analysis of chromatin interactions. Nature.

[7] Rao S.S., Huntley M.H., Durand N.C., Stamenova E.K., Bochkov I.D., Robinson J.T., Sanborn A.L., Machol I., Omer A.D., Lander E.S. (2014). A 3D map of the human genome at kilobase resolution reveals principles of chromatin looping. Cell.

[8] Blackwood E.M., Kadonaga J.T. (1998). Going the distance: A current view of enhancer action. Science.

[9] Nishioka N., Yamamoto S., Kiyonari H., Sato H., Sawada A., Ota M., Nakao K., Sasaki H. (2008). TEAD4 is required for specification of trophectoderm in pre-implantation mouse embryos. Mech. Dev..

[10] Yagi R., Kohn M.J., Karavanova I., Kaneko K.J., Vullhorst D., DePamphilis M.L., Buonanno A. (2007). Transcription factor TEAD4 specifies the trophectoderm lineage at the beginning of mammalian development. Development.

[11] Wicklow E., Blij S., Frum T., Hirate Y., Lang R.A., Sasaki H., Ralston A. (2014). HIPPO pathway members restrict SOX2 to the inner cell mass where it promotes icm fates in the mouse blastocyst. PLoS Genet..

[12] Avilion A.A., Nicolis S.K., Pevny L.H., Perez L., Vivian N., Lovell-Badge R. (2003). Multipotent cell lineages in early mouse development depend on SOX2 function. Genes Dev..

[13] Aksoy I., Jauch R., Chen J., Dyla M., Divakar U., Bogu G.K., Teo R., Leng Ng C.K., Herath W., Lili S. (2013). OCT4 switches partnering from SOX2 to SOX17 to reinterpret the enhancer code and specify endoderm. EMBO J..

[14] Chen X., Xu H., Yuan P., Fang F., Huss M., Vega V.B., Wong E., Orlov Y.L., Zhang W., Jiang J. (2008). Integration of external signaling pathways with the core transcriptional network in embryonic stem cells. Cell.

[15] Nichols J., Zevnik B., Anastassiadis K., Niwa H., Klewe-Nebenius D., Chambers I., Scholer H., Smith A. (1998). Formation of pluripotent stem cells in the mammalian embryo depends on the POU transcription factor OCT4. Cell.

[16] Plachta N., Bollenbach T., Pease S., Fraser S.E., Pantazis P. (2011). OCT4 kinetics predict cell lineage patterning in the early mammalian embryo. Nat. Cell Biol..

[17] Kaur G., Costa M.W., Nefzger C.M., Silva J., Fierro-Gonzalez J.C., Polo J.M., Bell T.D., Plachta N. (2013). Probing transcription factor diffusion dynamics in the living mammalian embryo with photoactivatable fluorescence correlation spectroscopy. Nat. Commun..

[18] Mitsui K., Tokuzawa Y., Itoh H., Segawa K., Murakami M., Takahashi K., Maruyama M., Maeda M., Yamanaka S. (2003). The homeoprotein Nanog is required for maintenance of pluripotency in mouse epiblast and ES cells. Cell.

[19] Loh K.M., Lim B. (2011). A precarious balance: Pluripotency factors as lineage specifiers. Cell Stem Cell.

[20] Hussein S.M., Puri M.C., Tonge P.D., Benevento M., Corso A.J., Clancy J.L., Mosbergen R., Li M., Lee D.S., Cloonan N. (2014). Genome-wide characterization of the routes to pluripotency. Nature.

[21] Buganim Y., Faddah D.A., Cheng A.W., Itskovich E., Markoulaki S., Ganz K., Klemm S.L., van Oudenaarden A., Jaenisch R. (2012). Single-cell expression analyses during cellular reprogramming reveal an early stochastic and a late hierarchic phase. Cell.

[22] Feng B., Jiang J., Kraus P., Ng J.H., Heng J.C., Chan Y.S., Yaw L.P., Zhang W., Loh Y.H., Han J. (2009). Reprogramming of fibroblasts into induced pluripotent stem cells with orphan nuclear receptor esrrb. Nat. Cell Biol..

[23] Yu J., Vodyanik M.A., Smuga-Otto K., Antosiewicz-Bourget J., Frane J.L., Tian S., Nie J., Jonsdottir G.A., Ruotti V., Stewart R. (2007). Induced pluripotent stem cell lines derived from human somatic cells. Science.

[24] Shu J., Zhang K., Zhang M., Yao A., Shao S., Du F., Yang C., Chen W., Wu C., Yang W. (2015). Gata family members as inducers for cellular reprogramming to pluripotency. Cell Res..

[25] Schwarz B.A., Bar-Nur O., Silva J.C., Hochedlinger K. (2014). *Nanog* is dispensable for the generation of induced pluripotent stem cells. Curr. Biol..

[26] Hiratani I., Ryba T., Itoh M., Rathjen J., Kulik M., Papp B., Fussner E., Bazett-Jones D.P., Plath K., Dalton S. (2010). Genome-wide dynamics of replication timing revealed by *in vitro* models of mouse embryogenesis. Genome Res..

[27] Ricci M.A., Manzo C., Garcia-Parajo M.F., Lakadamyali M., Cosma M.P. (2015). Chromatin fibers are formed by heterogeneous groups of nucleosomes *in vivo*. Cell.

[28] Fan Y., Nikitina T., Zhao J., Fleury T.J., Bhattacharyya R., Bouhassira E.E., Stein A., Woodcock C.L., Skoultchi A.I. (2005). Histone H1 depletion in mammals alters global chromatin structure but causes specific changes in gene regulation. Cell.

[29] Marson A., Levine S.S., Cole M.F., Frampton G.M., Brambrink T., Johnstone S., Guenther M.G., Johnston W.K., Wernig M., Newman J. (2008). Connecting microRNA genes to the core transcriptional regulatory circuitry of embryonic stem cells. Cell.

[30] Mikkelsen T.S., Ku M., Jaffe D.B., Issac B., Lieberman E., Giannoukos G., Alvarez P., Brockman W., Kim T.K., Koche R.P. (2007). Genome-wide maps of chromatin state in pluripotent and lineage-committed cells. Nature.

[31] Heintzman N.D., Stuart R.K., Hon G., Fu Y., Ching C.W., Hawkins R.D., Barrera L.O., van Calcar S., Qu C., Ching K.A. (2007). Distinct and predictive chromatin signatures of transcriptional promoters and enhancers in the human genome. Nat. Genet..

[32] Ferrari K.J., Scelfo A., Jammula S., Cuomo A., Barozzi I., Stutzer A., Fischle W., Bonaldi T., Pasini D. (2014). Polycomb-dependent H3K27me1 and H3K27me2 regulate active transcription and enhancer fidelity. Mol. Cell.

[33] Denholtz M., Bonora G., Chronis C., Splinter E., de Laat W., Ernst J., Pellegrini M., Plath K. (2013). Long-range chromatin contacts in embryonic stem cells reveal a role for pluripotency factors and polycomb proteins in genome organization. Cell Stem Cell.

[34] Czermin B., Melfi R., McCabe D., Seitz V., Imhof A., Pirrotta V. (2002). Drosophila enhancer of Zeste/ESC complexes have a histone H3 methyltransferase activity that marks chromosomal Polycomb sites. Cell.

[35] Peters A.H., Kubicek S., Mechtler K., O’Sullivan R.J., Derijck A.A., Perez-Burgos L., Kohlmaier A., Opravil S., Tachibana M., Shinkai Y. (2003). Partitioning and plasticity of repressive histone methylation states in mammalian chromatin. Mol. Cell.

[36] Fussner E., Djuric U., Strauss M., Hotta A., Perez-Iratxeta C., Lanner F., Dilworth F.J., Ellis J., Bazett-Jones D.P. (2011). Constitutive heterochromatin reorganization during somatic cell reprogramming. EMBO J..

[37] Ostuni R., Piccolo V., Barozzi I., Polletti S., Termanini A., Bonifacio S., Curina A., Prosperini E., Ghisletti S., Natoli G. (2013). Latent enhancers activated by stimulation in differentiated cells. Cell.

[38] Heintzman N.D., Hon G.C., Hawkins R.D., Kheradpour P., Stark A., Harp L.F., Ye Z., Lee L.K., Stuart R.K., Ching C.W. (2009). Histone modifications at human enhancers reflect global cell-type-specific gene expression. Nature.

[39] Bernstein B.E., Kamal M., Lindblad-Toh K., Bekiranov S., Bailey D.K., Huebert D.J., McMahon S., Karlsson E.K., Kulbokas E.J., Gingeras T.R. (2005). Genomic maps and comparative analysis of histone modifications in human and mouse. Cell.

[40] Visel A., Blow M.J., Li Z., Zhang T., Akiyama J.A., Holt A., Plajzer-Frick I., Shoukry M., Wright C., Chen F. (2009). CHIP-seq accurately predicts tissue-specific activity of enhancers. Nature.

[41] Rada-Iglesias A., Bajpai R., Swigut T., Brugmann S.A., Flynn R.A., Wysocka J. (2011). A unique chromatin signature uncovers early developmental enhancers in humans. Nature.

[42] Chen C.Y., Morris Q., Mitchell J.A. (2012). Enhancer identification in mouse embryonic stem cells using integrative modeling of chromatin and genomic features. BMC Genomics.

[43] Kerppola T.K. (2009). Polycomb group complexes—Many combinations, many functions. Trends Cell Biol..

[44] Creyghton M.P., Cheng A.W., Welstead G.G., Kooistra T., Carey B.W., Steine E.J., Hanna J., Lodato M.A., Frampton G.M., Sharp P.A. (2010). Histone H3K27ac separates active from poised enhancers and predicts developmental state. Proc. Natl. Acad. Sci. USA.

[45] Zentner G.E., Tesar P.J., Scacheri P.C. (2011). Epigenetic signatures distinguish multiple classes of enhancers with distinct cellular functions. Genome Res..

[46] Mikkelsen T.S., Hanna J., Zhang X., Ku M., Wernig M., Schorderet P., Bernstein B.E., Jaenisch R., Lander E.S., Meissner A. (2008). Dissecting direct reprogramming through integrative genomic analysis. Nature.

[47] Zhao X.Y., Li W., Lv Z., Liu L., Tong M., Hai T., Hao J., Guo C.L., Ma Q.W., Wang L. (2009). iPS cells produce viable mice through tetraploid complementation. Nature.

[48] Mansour A.A., Gafni O., Weinberger L., Zviran A., Ayyash M., Rais Y., Krupalnik V., Zerbib M., Amann-Zalcenstein D., Maza I. (2012). The H3K27 demethylase Utx regulates somatic and germ cell epigenetic reprogramming. Nature.

[49] Koche R.P., Smith Z.D., Adli M., Gu H., Ku M., Gnirke A., Bernstein B.E., Meissner A. (2011). Reprogramming factor expression initiates widespread targeted chromatin remodeling. Cell Stem Cell.

[50] Samavarchi-Tehrani P., Golipour A., David L., Sung H.K., Beyer T.A., Datti A., Woltjen K., Nagy A., Wrana J.L. (2010). Functional genomics reveals a bmp-driven mesenchymal-to-epithelial transition in the initiation of somatic cell reprogramming. Cell Stem Cell.

[51] Lee D.S., Shin J.Y., Tonge P.D., Puri M.C., Lee S., Park H., Lee W.C., Hussein S.M., Bleazard T., Yun J.Y. (2014). An epigenomic roadmap to induced pluripotency reveals DNA methylation as a reprogramming modulator. Nat. Commun..

[52] Li G., Ruan X., Auerbach R.K., Sandhu K.S., Zheng M., Wang P., Poh H.M., Goh Y., Lim J., Zhang J. (2012). Extensive promoter-centered chromatin interactions provide a topological basis for transcription regulation. Cell.

[53] Phillips-Cremins J.E., Sauria M.E., Sanyal A., Gerasimova T.I., Lajoie B.R., Bell J.S., Ong C.T., Hookway T.A., Guo C., Sun Y. (2013). Architectural protein subclasses shape 3D organization of genomes during lineage commitment. Cell.

[54] Wang Z., Oron E., Nelson B., Razis S., Ivanova N. (2012). Distinct lineage specification roles for Nanog, OCT4, and SOX2 in human embryonic stem cells. Cell Stem Cell.

[55] Bulger M., Groudine M. (2010). Enhancers: The abundance and function of regulatory sequences beyond promoters. Dev. Biol..

[56] Hallikas O., Palin K., Sinjushina N., Rautiainen R., Partanen J., Ukkonen E., Taipale J. (2006). Genome-wide prediction of mammalian enhancers based on analysis of transcription-factor binding affinity. Cell.

[57] Hnisz D., Abraham B.J., Lee T.I., Lau A., Saint-Andre V., Sigova A.A., Hoke H.A., Young R.A. (2013). Super-enhancers in the control of cell identity and disease. Cell.

[58] Pott S., Lieb J.D. (2015). What are super-enhancers?. Nat. Genet..

[59] Zhou H.Y., Katsman Y., Dhaliwal N.K., Davidson S., Macpherson N.N., Sakthidevi M., Collura F., Mitchell J.A. (2014). A SOX2 distal enhancer cluster regulates embryonic stem cell differentiation potential. Genes Dev..

[60] Li Y., Rivera C.M., Ishii H., Jin F., Selvaraj S., Lee A.Y., Dixon J.R., Ren B. (2014). Crispr reveals a distal super-enhancer required for SOX2 expression in mouse embryonic stem cells. PLoS ONE.

[61] Heinz S., Benner C., Spann N., Bertolino E., Lin Y.C., Laslo P., Cheng J.X., Murre C., Singh H., Glass C.K. (2010). Simple combinations of lineage-determining transcription factors prime cis-regulatory elements required for macrophage and b cell identities. Mol. Cell.

[62] Thurman R.E., Rynes E., Humbert R., Vierstra J., Maurano M.T., Haugen E., Sheffield N.C., Stergachis A.B., Wang H., Vernot B. (2012). The accessible chromatin landscape of the human genome. Nature.

[63] Natoli G. (2010). Maintaining cell identity through global control of genomic organization. Immunity.

[64] Odom D.T., Zizlsperger N., Gordon D.B., Bell G.W., Rinaldi N.J., Murray H.L., Volkert T.L., Schreiber J., Rolfe P.A., Gifford D.K. (2004). Control of pancreas and liver gene expression by HNF transcription factors. Science.

[65] Rosenbauer F., Wagner K., Kutok J.L., Iwasaki H., le Beau M.M., Okuno Y., Akashi K., Fiering S., Tenen D.G. (2004). Acute myeloid leukemia induced by graded reduction of a lineage-specific transcription factor, PU.1. Nat. Genet..

[66] Zhu J., Bennett C.A., MacGregor A.D., Greaves M.F., Goodwin G.H., Ford A.M. (1994). A myeloid-lineage-specific enhancer upstream of the mouse myeloperoxidase (MPO) gene. Leukemia.

[67] Chandler K.J., Chandler R.L., Mortlock D.P. (2009). Identification of an ancient BMP4 mesoderm enhancer located 46 kb from the promoter. Dev. Biol..

[68] Papanayotou C., Benhaddou A., Camus A., Perea-Gomez A., Jouneau A., Mezger V., Langa F., Ott S., Saberan-Djoneidi D., Collignon J. (2014). A novel nodal enhancer dependent on pluripotency factors and Smad2/3 signaling conditions a regulatory switch during epiblast maturation. PLoS Biol..

[69] Kang J., Nathan E., Xu S.M., Tzahor E., Black B.L. (2009). Isl1 is a direct transcriptional target of forkhead transcription factors in second-heart-field-derived mesoderm. Dev. Biol..

[70] Decker T., Pasca di Magliano M., McManus S., Sun Q., Bonifer C., Tagoh H., Busslinger M. (2009). Stepwise activation of enhancer and promoter regions of the B cell commitment gene Pax5 in early lymphopoiesis. Immunity.

[71] Pfeffer P.L., Bouchard M., Busslinger M. (2000). Pax2 and homeodomain proteins cooperatively regulate a 435 bp enhancer of the mouse Pax5 gene at the midbrain-hindbrain boundary. Development.

[72] Gustafsson M.K., Pan H., Pinney D.F., Liu Y., Lewandowski A., Epstein D.J., Emerson C.P. (2002). Myf5 is a direct target of long-range Shh signaling and Gli regulation for muscle specification. Genes Dev..

[73] Teboul L., Hadchouel J., Daubas P., Summerbell D., Buckingham M., Rigby P.W. (2002). The early epaxial enhancer is essential for the initial expression of the skeletal muscle determination gene Myf5 but not for subsequent, multiple phases of somitic myogenesis. Development.

[74] Shirai T., Miyagi S., Horiuchi D., Okuda-Katayanagi T., Nishimoto M., Muramatsu M., Sakamoto Y., Nagata M., Hagiwara K., Okuda A. (2005). Identification of an enhancer that controls up-regulation of fibronectin during differentiation of embryonic stem cells into extraembryonic endoderm. J. Biol. Chem..

[75] Dodou E., Verzi M.P., Anderson J.P., Xu S.M., Black B.L. (2004). Mef2c is a direct transcriptional target of isl1 and gata factors in the anterior heart field during mouse embryonic development. Development.

[76] Werner T., Hammer A., Wahlbuhl M., Bosl M.R., Wegner M. (2007). Multiple conserved regulatory elements with overlapping functions determine SOX10 expression in mouse embryogenesis. Nucleic Acids Res..

[77] Kuspert M., Hammer A., Bosl M.R., Wegner M. (2011). Olig2 regulates SOX10 expression in oligodendrocyte precursors through an evolutionary conserved distal enhancer. Nucleic Acids Res..

[78] Carbe C., Hertzler-Schaefer K., Zhang X. (2012). The functional role of the meis/prep-binding elements in pax6 locus during pancreas and eye development. Dev. Biol..

[79] Kleinjan D.A., Seawright A., Mella S., Carr C.B., Tyas D.A., Simpson T.I., Mason J.O., Price D.J., van Heyningen V. (2006). Long-range downstream enhancers are essential for Pax6 expression. Dev. Biol..

[80] Bhatia S., Monahan J., Ravi V., Gautier P., Murdoch E., Brenner S., van Heyningen V., Venkatesh B., Kleinjan D.A. (2014). A survey of ancient conserved non-coding elements in the Pax6 locus reveals a landscape of interdigitated *cis*-regulatory archipelagos. Dev. Biol..

[81] Drissen R., Guyot B., Zhang L., Atzberger A., Sloane-Stanley J., Wood B., Porcher C., Vyas P. (2010). Lineage-specific combinatorial action of enhancers regulates mouse erythroid gata1 expression. Blood.

[82] Wang S., Sengel C., Emerson M.M., Cepko C.L. (2014). A gene regulatory network controls the binary fate decision of rod and bipolar cells in the vertebrate retina. Dev. Cell.

[83] Samstein R.M., Arvey A., Josefowicz S.Z., Peng X., Reynolds A., Sandstrom R., Neph S., Sabo P., Kim J.M., Liao W. (2012). Foxp3 exploits a pre-existent enhancer landscape for regulatory T cell lineage specification. Cell.

[84] Papanayotou C., Mey A., Birot A.M., Saka Y., Boast S., Smith J.C., Samarut J., Stern C.D. (2008). A mechanism regulating the onset of SOX2 expression in the embryonic neural plate. PLoS Biol..

[85] Wang A., Yue F., Li Y., Xie R., Harper T., Patel N.A., Muth K., Palmer J., Qiu Y., Wang J. (2015). Epigenetic priming of enhancers predicts developmental competence of hESC-derived endodermal lineage intermediates. Cell Stem Cell.

[86] Soufi A., Donahue G., Zaret K.S. (2012). Facilitators and impediments of the pluripotency reprogramming factors’ initial engagement with the genome. Cell.

[87] Taberlay P.C., Kelly T.K., Liu C.C., You J.S., de Carvalho D.D., Miranda T.B., Zhou X.J., Liang G., Jones P.A. (2011). Polycomb-repressed genes have permissive enhancers that initiate reprogramming. Cell.

[88] Gao X., Yang J., Tsang J.C., Ooi J., Wu D., Liu P. (2013). Reprogramming to pluripotency using designer TALE transcription factors targeting enhancers. Stem Cell Rep..

[89] Gao X., Tsang J.C., Gaba F., Wu D., Lu L., Liu P. (2014). Comparison of TALE designer transcription factors and the CRISPR/dCas9 in regulation of gene expression by targeting enhancers. Nucleic Acids Res..

[90] Ahmed K., Dehghani H., Rugg-Gunn P., Fussner E., Rossant J., Bazett-Jones D.P. (2010). Global chromatin architecture reflects pluripotency and lineage commitment in the early mouse embryo. PLoS ONE.

[91] Kurisaki A., Hamazaki T.S., Okabayashi K., Iida T., Nishine T., Chonan R., Kido H., Tsunasawa S., Nishimura O., Asashima M. (2005). Chromatin-related proteins in pluripotent mouse embryonic stem cells are downregulated after removal of leukemia inhibitory factor. Biochem. Biophys. Res. Commun..

[92] Efroni S., Duttagupta R., Cheng J., Dehghani H., Hoeppner D.J., Dash C., Bazett-Jones D.P., le Grice S., McKay R.D., Buetow K.H. (2008). Global transcription in pluripotent embryonic stem cells. Cell Stem Cell.

[93] Faro-Trindade I., Cook P.R. (2006). A conserved organization of transcription during embryonic stem cell differentiation and in cells with high c value. Mol. Biol. Cell.

[94] Ghamari A., van de Corput M.P., Thongjuea S., van Cappellen W.A., van Ijcken W., van Haren J., Soler E., Eick D., Lenhard B., Grosveld F.G. (2013). *In vivo* live imaging of RNA polymerase II transcription factories in primary cells. Genes Dev..

[95] Cisse I.I., Izeddin I., Causse S.Z., Boudarene L., Senecal A., Muresan L., Dugast-Darzacq C., Hajj B., Dahan M., Darzacq X. (2013). Real-time dynamics of RNA polymerase II clustering in live human cells. Science.

[96] Meshorer E., Misteli T. (2006). Chromatin in pluripotent embryonic stem cells and differentiation. Nat. Rev. Mol. Cell Biol..

[97] Hinde E., Cardarelli F., Chen A., Khine M., Gratton E. (2012). Tracking the mechanical dynamics of human embryonic stem cell chromatin. Epigenetics Chromatin.

[98] Park S.H., Park S.H., Kook M.C., Kim E.Y., Park S., Lim J.H. (2004). Ultrastructure of human embryonic stem cells and spontaneous and retinoic acid-induced differentiating cells. Ultrastruct. Pathol..

[99] Parada L.A., McQueen P.G., Misteli T. (2004). Tissue-specific spatial organization of genomes. Genome Biol..

[100] Smeets D., Markaki Y., Schmid V.J., Kraus F., Tattermusch A., Cerase A., Sterr M., Fiedler S., Demmerle J., Popken J. (2014). Three-dimensional super-resolution microscopy of the inactive X chromosome territory reveals a collapse of its active nuclear compartment harboring distinct Xist RNA foci. Epigenetics Chromatin.

[101] Guyochin A., Maenner S., Chu E.T., Hentati A., Attia M., Avner P., Clerc P. (2014). Live cell imaging of the nascent inactive x chromosome during the early differentiation process of naive es cells towards epiblast stem cells. PLoS ONE.

[102] Pasque V., Tchieu J., Karnik R., Uyeda M., Sadhu Dimashkie A., Case D., Papp B., Bonora G., Patel S., Ho R. (2014). X chromosome reactivation dynamics reveal stages of reprogramming to pluripotency. Cell.

[103] Dekker J., Rippe K., Dekker M., Kleckner N. (2002). Capturing chromosome conformation. Science.

[104] Lieberman-Aiden E., van Berkum N.L., Williams L., Imakaev M., Ragoczy T., Telling A., Amit I., Lajoie B.R., Sabo P.J., Dorschner M.O. (2009). Comprehensive mapping of long-range interactions reveals folding principles of the human genome. Science.

[105] Williams R.R., Broad S., Sheer D., Ragoussis J. (2002). Subchromosomal positioning of the epidermal differentiation complex (EDC) in keratinocyte and lymphoblast interphase nuclei. Exp. Cell Res..

[106] Osborne C.S., Chakalova L., Brown K.E., Carter D., Horton A., Debrand E., Goyenechea B., Mitchell J.A., Lopes S., Reik W. (2004). Active genes dynamically colocalize to shared sites of ongoing transcription. Nat. Genet..

[107] Schoenfelder S., Sexton T., Chakalova L., Cope N.F., Horton A., Andrews S., Kurukuti S., Mitchell J.A., Umlauf D., Dimitrova D.S. (2010). Preferential associations between co-regulated genes reveal a transcriptional interactome in erythroid cells. Nat. Genet..

[108] Boyle S., Rodesch M.J., Halvensleben H.A., Jeddeloh J.A., Bickmore W.A. (2011). Fluorescence *in situ* hybridization with high-complexity repeat-free oligonucleotide probes generated by massively parallel synthesis. Chromosome Res..

[109] Pope B.D., Ryba T., Dileep V., Yue F., Wu W., Denas O., Vera D.L., Wang Y., Hansen R.S., Canfield T.K. (2014). Topologically associating domains are stable units of replication-timing regulation. Nature.

[110] Simonis M., Klous P., Splinter E., Moshkin Y., Willemsen R., de Wit E., van Steensel B., de Laat W. (2006). Nuclear organization of active and inactive chromatin domains uncovered by chromosome conformation capture-on-chip (4c). Nat. Genet..

[111] Dostie J., Richmond T.A., Arnaout R.A., Selzer R.R., Lee W.L., Honan T.A., Rubio E.D., Krumm A., Lamb J., Nusbaum C. (2006). Chromosome conformation capture carbon copy (5c): A massively parallel solution for mapping interactions between genomic elements. Genome Res..

[112] Fullwood M.J., Liu M.H., Pan Y.F., Liu J., Xu H., Mohamed Y.B., Orlov Y.L., Velkov S., Ho A., Mei P.H. (2009). An oestrogen-receptor-alpha-bound human chromatin interactome. Nature.

[113] Hughes J.R., Roberts N., McGowan S., Hay D., Giannoulatou E., Lynch M., de Gobbi M., Taylor S., Gibbons R., Higgs D.R. (2014). Analysis of hundreds of *cis*-regulatory landscapes at high resolution in a single, high-throughput experiment. Nat. Genet..

[114] Schoenfelder S., Furlan-Magaril M., Mifsud B., Tavares-Cadete F., Sugar R., Javierre B.M., Nagano T., Katsman Y., Sakthidevi M., Wingett S.W. (2015). The pluripotent regulatory circuitry connecting promoters to their long-range interacting elements. Genome Res..

[115] Kagey M.H., Newman J.J., Bilodeau S., Zhan Y., Orlando D.A., van Berkum N.L., Ebmeier C.C., Goossens J., Rahl P.B., Levine S.S. (2010). Mediator and cohesin connect gene expression and chromatin architecture. Nature.

[116] Heath H., Ribeiro de Almeida C., Sleutels F., Dingjan G., van de Nobelen S., Jonkers I., Ling K.W., Gribnau J., Renkawitz R., Grosveld F. (2008). Ctcf regulates cell cycle progression of alphabeta T cells in the thymus. EMBO J..

[117] Chia N.Y., Chan Y.S., Feng B., Lu X., Orlov Y.L., Moreau D., Kumar P., Yang L., Jiang J., Lau M.S. (2010). A genome-wide rnai screen reveals determinants of human embryonic stem cell identity. Nature.

[118] Heidari N., Phanstiel D.H., He C., Grubert F., Jahanbani F., Kasowski M., Zhang M.Q., Snyder M.P. (2014). Genome-wide map of regulatory interactions in the human genome. Genome Res..

[119] Bailey S.D., Zhang X., Desai K., Aid M., Corradin O., Cowper-Sal Lari R., Akhtar-Zaidi B., Scacheri P.C., Haibe-Kains B., Lupien M. (2015). ZNF143 provides sequence specificity to secure chromatin interactions at gene promoters. Nat. Commun..

[120] Chen X., Fang F., Liou Y.C., Ng H.H. (2008). ZFP143 regulates nanog through modulation of OCT4 binding. Stem Cells.

[121] Wei Z., Gao F., Kim S., Yang H., Lyu J., An W., Wang K., Lu W. (2013). Klf4 organizes long-range chromosomal interactions with the oct4 locus in reprogramming and pluripotency. Cell Stem Cell.

[122] Liu Z., Scannell D.R., Eisen M.B., Tjian R. (2011). Control of embryonic stem cell lineage commitment by core promoter factor, TAF3. Cell.

[123] Abboud N., Morris T.M., Hiriart E., Yang H., Bezerra H., Gualazzi M.G., Stefanovic S., Guenantin A.C., Evans S.M., Puceat M. (2015). A cohesin-OCT4 complex mediates Sox enhancers to prime an early embryonic lineage. Nat. Commun..

[124] Zhang Y., Wong C.H., Birnbaum R.Y., Li G., Favaro R., Ngan C.Y., Lim J., Tai E., Poh H.M., Wong E. (2013). Chromatin connectivity maps reveal dynamic promoter-enhancer long-range associations. Nature.

[125] Apostolou E., Hochedlinger K. (2013). Chromatin dynamics during cellular reprogramming. Nature.

[126] Apostolou E., Ferrari F., Walsh R.M., Bar-Nur O., Stadtfeld M., Cheloufi S., Stuart H.T., Polo J.M., Ohsumi T.K., Borowsky M.L. (2013). Genome-wide chromatin interactions of the Nanog locus in pluripotency, differentiation, and reprogramming. Cell Stem Cell.

[127] Zhang H., Jiao W., Sun L., Fan J., Chen M., Wang H., Xu X., Shen A., Li T., Niu B. (2013). Intrachromosomal looping is required for activation of endogenous pluripotency genes during reprogramming. Cell Stem Cell.

[128] Nagano T., Lubling Y., Stevens T.J., Schoenfelder S., Yaffe E., Dean W., Laue E.D., Tanay A., Fraser P. (2013). Single-cell Hi-C reveals cell-to-cell variability in chromosome structure. Nature.

[129] Mali P., Yang L., Esvelt K.M., Aach J., Guell M., DiCarlo J.E., Norville J.E., Church G.M. (2013). RNA-guided human genome engineering via Cas9. Science.

[130] Qi L.S., Larson M.H., Gilbert L.A., Doudna J.A., Weissman J.S., Arkin A.P., Lim W.A. (2013). Repurposing CRISPR as an RNA-guided platform for sequence-specific control of gene expression. Cell.

[131] Gilbert L.A., Larson M.H., Morsut L., Liu Z., Brar G.A., Torres S.E., Stern-Ginossar N., Brandman O., Whitehead E.H., Doudna J.A. (2013). CRISPR-mediated modular RNA-guided regulation of transcription in eukaryotes. Cell.

[132] Hilton I.B., D’Ippolito A.M., Vockley C.M., Thakore P.I., Crawford G.E., Reddy T.E., Gersbach C.A. (2015). Epigenome editing by a CRISPR-Cas9-based acetyltransferase activates genes from promoters and enhancers. Nat. Biotechnol..

[133] Kearns N.A., Pham H., Tabak B., Genga R.M., Silverstein N.J., Garber M., Maehr R. (2015). Functional annotation of native enhancers with a Cas9-histone demethylase fusion. Nat. Methods.

[134] Deng W., Rupon J.W., Krivega I., Breda L., Motta I., Jahn K.S., Reik A., Gregory P.D., Rivella S., Dean A. (2014). Reactivation of developmentally silenced globin genes by forced chromatin looping. Cell.

